# Supplementary material for: A Bacterial Cell-Based Assay To Study SARS-CoV-2 Protein-Protein Interactions
Source: mBio. 2021 Nov 16;12(6):e02936-21. doi: 10.1128/mBio.02936-21 (PMC8593686; doi:10.1128/mBio.02936-21)
Supplement: TABLE S1 [file mbio.02936-21-st001.pdf]

Supplementary Information

Table S1A: Strains and plasmids

Strain	Details	Reference
MAX Efficiency™ DH5αF‘IQ	F-φ80lacZΔM15 Δ(lacZYA-argF) U169 recA1 endA1 hsdR17 (rk-, mk+) phoA supE44 λ-thi-1 gyrA96 relA1/F´ proAB+ lacIqZΔM15 zzf::Tn5. Used for cloning into pBRωGP. (KmR)	Invitrogen
NEB® 5-alpha F‘IQ	F‘ *proA*$^+$*B*$^+$ *lacI*^{q} *Δ(lacZ)M15 zzf::Tn10* (Tet^R) */ fhuA2Δ(argF-lacZ)U169 phoA glnV44 Φ80Δ(lacZ)M15 gyrA96 recA1 relA1 endA1 thi-1 hsdR17*. Used for cloning into pBRα and pACλCI vectors. (TetR)	New England Biolabs (NEB)
FW102 O$_L$2–62	Classical bacterial two-hybrid reporter strain. FW102 containing an F' bearing the P*lac*O$_L$2-62-*lacZ* fusion with the λCI operator at position -62 upstream of the transcription start site (KmR)	[1]
SHuffle® Express	Strain for non-T7 protein expression with enhanced likelihood for correctly folded disulfide bond-containing proteins in the *E. coli* cytoplasm. (SpR)	NEB
BLS128	SHuffle® Express, Δ*lacIZYA::kmR*	This study
BLS133	SHuffle® Express, Δ*lacIZYA*	This study
BLS148	Modified bacterial two-hybrid reporter strain that allows disulfide bond formation in the *E. coli* cytoplasm. SHuffle® Express, Δ*lacIZYA* containing an F' bearing the P*lac*O$_L$2-62-*lacZ* fusion with the λCI operator at position -62 upstream of the transcription start site (KmR, SpR)	This study

Plasmids	Details	Reference
pACλCI	P$_{lacUV5}$-directed synthesis of the λCI protein. (CmR)	[2]
pACλCI-β-flap	P$_{lacUV5}$-directed synthesis of the λCI protein fused via three alanines to residues 831-1057 of the β subunit of *E. coli* RNAP. Used as positive control together with pBRα-σ^{70} D581G	[3]
pBRα	P$_{lacUV5}$-directed synthesis of the full length α subunit of *E. coli* RNAP. (AmpR)	[2]

pBRα-σ^70^ D581G	P_{lacUV5}-directed synthesis of the αNTD (residues 1-248 of the α subunit of *E. coli* RNAP) fused directly to *E. coli* σ^{70} region 4 (residues 528-613 of σ^{70}). The σ^{70} moiety also carries the D581G substitution. Used as positive control together with pACλCI-β-flap.	[4]
pBRωGP	P_{lacUV5}-directed synthesis of Gal11P fused to the C-terminus of *E. coli* RNAP subunit ω.	[5]
pS53	pBRα-3a	This study
pS57	pACλCI-3a	This study
pS63	pBRα-S, aa 1-1273	This study
pS64	pBRα-S (NTD), aa 1-330	This study
pS65	pBRα-S (RBD), aa 331-521	This study
pS66	pBRα-S (CTD), aa 522-1273	This study
pS67	pBRα-S (Ecto-13), aa 13-1213	This study
pS68	pBRα-S (Ecto-16), aa 16-1213	This study
pS70	pBRα-S (CTD without TMD), aa 522-1213	This study
pS72	pACλCI-S, aa 1-1273	This study
pS73	pACλCI-S (NTD), aa 1-330	This study
pS74	pACλCI-S (RBD), aa 331-521	This study
pS75	pACλCI-S (CTD), aa 522-1273	This study
pS76	pACλCI-S (Ecto-13), aa 13-1213	This study
pS77	pACλCI-S (Ecto-16), aa 16-1213	This study
pS79	pACλCI-S (CTD without TMD), aa 522-1213	This study
pS85	pBRω-Nsp2	This study
pS89	pBRω-Nsp3	This study
pS169	pBRω-helicase (Nsp13)	This study
pS173	pBRω-RNA-Polymerase (Nsp12)	This study
pS177	pBRα-Nsp1	This study
pS178	pACλCI-Nsp1	This study
pS179	pBRα-Nsp2	This study
pS180	pACλCI-Nsp2	This study
pS181	pBRα-Nsp3	This study
pS182	pACλCI-Nsp3	This study
pS183	pBRα-Nsp4	This study
pS184	pACλCI-Nsp4	This study

pS185	pBRα-Nsp5	This study
pS186	pACλCI-Nsp5	This study
pS187	pBRα-Nsp6	This study
pS188	pACλCI-Nsp6	This study
pS189	pBRα-Nsp7	This study
pS190	pACλCI-Nsp7	This study
pS191	pBRα-Nsp8	This study
pS192	pACλCI-Nsp8	This study
pS193	pBRα-Nsp9	This study
pS194	pACλCI-Nsp9	This study
pS195	pBRα-Nsp10	This study
pS196	pACλCI-Nsp10	This study
pS197	pBRα-Nsp11	This study
pS198	pACλCI-Nsp11	This study
pS199	pBRα-ORF6	This study
pS200	pACλCI-ORF6	This study
pS201	pBRα-ORF7a	This study
pS202	pACλCI-ORF7a	This study
pS203	pBRα-ORF7b	This study
pS204	pACλCI-ORF7b	This study
pS205	pBRα-ORF8	This study
pS206	pACλCI-ORF8	This study
pS207	pBRα-ORF10	This study
pS208	pACλCI-ORF10	This study
pS209	pBRα-M	This study
pS210	pACλCI-M	This study
pS211	pBRα-N	This study
pS212	pACλCI-N	This study
pS213	pBRα-E	This study
pS214	pACλCI-E	This study
pS215	pBRα-methyltransferase (Nsp16)	This study
pS216	pACλCI-methyltransferase (Nsp16)	This study
pS217	pBRα-endoRNase (Nsp15)	This study
pS218	pACλCI-endoRNase (Nsp15)	This study
pS219	pBRα-3’-5’-exonuclease (Nsp14)	This study

pS220	pACλCI-3’-5’-exonuclease (Nsp14)	This study
pS221	pBRα-helicase (Nsp13)	This study
pS222	pACλCI-helicase (Nsp13)	This study
pS223	pBRα-RNA-Polymerase (Nsp12)	This study
pS224	pACλCI-RNA-Polymerase (Nsp12)	This study
pS254	pBRα-methyltransferase (K76A+Q87A+D106A)	This study
pS256	pBRα-methyltransferase (I40A+M41A+V44A)	This study
pS257	pBRα-methyltransferase (I40A+M41A+V44A+K76A+Q87A+D106A)	This study
pS260	pBRα-ACE2 N-terminal peptidase domain, aa 19-615	This study
pS261	pACλCI-ACE2 N-terminal peptidase domain, aa 19-615	This study
pS262	pACλCI-Nsp10 (T5A+T12A+S15A)	This study
pS263	pACλCI-Nsp10 (D29A+S33A)	This study
pS264	pACλCI-Nsp10 (F16A+F19A+V21A)	This study
pS267	pBRα-S (RBD, N501Y)	This study
pS271	pBRα-S (RBD, C379S)	This study
pS272	pBRα- S (RBD, C432S)	This study
pS273	pBRα- S (RBD, C379S+C432S)	This study
pS275	pBRα- S (RBD, L452R)	This study
pS276	pBRα- S (RBD, E484K)	This study
pS277	pBRα- S (RBD, K417N)	This study
pS278	pBRα- S (RBD, N501Y+E484K)	This study
pS279	pBRα- S (RBD, N501Y+K417N)	This study
pS280	pBRα- S (RBD, N501Y+E484K+K417N)	This study

KmR: kanamycin resistance, CmR: chloramphenicol resistance, AmpR: ampicillin resistance, SpR: spectinomycin resistance, TMD: transmembrane domain

Table S1B: Oligonucleotides

Name	Sequence (5'->3')	Purpose
SARS_17	GGGCCATTTGATGTATTGTTCATACTTTCC	Cloning pS70, pS79
SARS_67	GAAAGAAGAGAAACCAGAGGCGGCCGCAATGTTCGTC	Cloning pS63, pS64
SARS_68	CACGATGCGTCCGGCGTAGAGGATCCTTACGTATAGTGTAACTTGACACCTTTCA	Cloning pS63, pS66
SARS_69	ACGATGCGTCCGGCGTAGAGGATCCTTACGGAAAGCGCAC	Cloning pS64
SARS_70	GAAAGAAGAGAAACCAGAGGCGGCCGCAAATATCACTAACC	Cloning pS65
SARS_71	ACGATGCGTCCGGCGTAGAGGATCCTTAGGGTGCGTGAAGC	Cloning pS65
SARS_72	GAAAGAAGAGAAACCAGAGGCGGCCGCAGCCACTGTGT	Cloning pS66
SARS_73	GAAAGAAGAGAAACCAGAGGCGGCCGCATCGCAATGTG	Cloning pS67
SARS_74	ACGATGCGTCCGGCGTAGAGGATCCTTAGGGCCATTTGATGTATTGT	Cloning pS67, pS68
SARS_75	GAAAGAAGAGAAACCAGAGGCGGCCGCAGTAAACTTGAC	Cloning pS68
SARS_76	TAAGGATCCTCTACGCCGGACG	Cloning pS70
SARS_77	CCTGAAGAGACGTTTGGCGCGGCCGCAATGTTCGTC	Cloning pS72, pS73
SARS_78	CGATGCAGATCTGTAAGGTAAGGATCCTTACGTATAGTGTAACTTGACACC	Cloning pS72, pS75
SARS_79	CGATGCAGATCTGTAAGGTAAGGATCCTTACGGAAAGCGCACG	Cloning pS73
SARS_80	GGCCTGAAGAGACGTTTGGCGCGGCCGCAAATATCACTAACC	Cloning pS74
SARS_81	CGATGCAGATCTGTAAGGTAAGGATCCTTAGGGTGCGTGAAGC	Cloning pS74
SARS_82	GGCCTGAAGAGACGTTTGGCGCGGCCGCAGCCACTGTGTG	Cloning pS75
SARS_83	GGCCTGAAGAGACGTTTGGCGCGGCCGCATCGCAATGTG	Cloning pS76
SARS_84	GATGCAGATCTGTAAGGTAAGGATCCTTAGGGCCATTTGATGTATTGT	Cloning pS76, pS77

SARS_85	GGCCTGAAGAGACGTTTGGCGCGGCCGCAGTAAACTTGAC	Cloning pS77
SARS_86	TAAGGATCCTTACCTTACAGATCTGCATCG	Cloning pS79
SARS_109	ATTGCTGAAGGTCGTCGTGCGGCCGCAGCTTATACAC	Cloning pS85
SARS_110	CCCTTAGTCACCTTGAAGTTGCCGCAGCTC	Cloning pS85
SARS_111	AACTTCAAGGTGACTAAGGGTAAGGCAAAGAAGG	Cloning pS85
SARS_112	GTTCTGCCAAGGCGCGCCGGATCCTCAGCCACCCTTCAG	Cloning pS85
SARS_115	TTGCTGAAGGTCGTCGTGCGGCCGCAGCACCCACG	Cloning pS89
SARS_116	GCGCAGTGCCTTGGCTAACATCTCGGTGGTTC	Cloning pS89
SARS_117	TGTTAGCCAAGGCACTGCGCAAGGTCCCT	Cloning pS89
SARS_118	CCGTGTTTCAGCTCGTATTGGGCGGGCGGG	Cloning pS89
SARS_119	CAATACGAGCTGAAACACGGGACCTTCACC	Cloning pS89
SARS_120	TCCCACTTGAAGCTGCTGATAGTGATCTGG	Cloning pS89
SARS_121	ATCAGCAGCTTCAAGTGGGATCTGACCGCATTTGG	Cloning pS89
SARS_122	TGTTCTGCCAAGGCGCGCCGGATCCTCAGCCGCCTTTCAG	Cloning pS89
SARS_125	TTGCTGAAGGTCGTCGTGCGGCCGCAGCAGTTGGC	Cloning pS169
SARS_126	CGCGAGCACTTGTCAATCGGTAAGTACTTAAGAG	Cloning pS169
SARS_127	TACCGATTGACAAGTGCTCGCGTATCATTCCGGCCC	Cloning pS169
SARS_128	TGTTCTGCCAAGGCGCGCCGGATCCTCACTGCAGGGTAGC	Cloning pS169
SARS_131	TTGCTGAAGGTCGTCGTGCGGCCGCATCAGCAGATG	Cloning pS173
SARS_132	GAAAATCTTGCGAACCAGCGGACCGAAGCTC	Cloning pS173
SARS_133	CGCTGGTTCGCAAGATTTTCGTGGATGGA	Cloning pS173
SARS_134	CCATCTCGCTCAGGACCTGGGCGCATTC	Cloning pS173
SARS_135	CCAGGTCCTGAGCGAGATGGTGATGTGCG	Cloning pS173
SARS_136	TTCTGCCAAGGCGCGCCGGGATCCTCATTGCAGAACGGTG	Cloning pS173

SARS_139	GGCCGCATCAGCTGATGCACAATCGTTTTTAAACGGGTTTGCGGTGTAAG	Cloning pS197, pS198
SARS_140	GATCCTTACACCGCAAACCCGTTTAAAAACGATTGTGCATCAGCTGATGC	Cloning pS197, pS198
SARS_253	CTGGGACTCTTTTGGTAGACTCGGACTTAAACGATTTCGTGTCGGCCGCCG ACTCGACACTCAT	Cloning pS254
SARS_254	TCGGCAGCCAGGCGCGGAGCACGGCGGTGCCCGGAGCTACCCCCGCGTCT GAGCCTGCGCCGAA	Cloning pS254
SARS_256	AACGCGGCGAAGTACACACAGTTGTGC	Cloning pS256, pS257
SARS_257	CATGGCCGCACCCTTTGGCAGAGTCGC	Cloning pS256, pS257
SARS_264	AAGAAGAGAAACCAGAGGCGGCCGCAAGCACCATCGAAGAACAGGC	Cloning pS260
SARS_265	GATGCGTCCGGCGTAGAGGATCCTTAATCCGCGTATGGAGACCAAT	Cloning pS260
SARS_266	CTGAAGAGACGTTTGGCGCGGCCGCAAGCACCATCGAAGAACAGGC	Cloning pS261
SARS_267	CGATGCAGATCTGTAAGGTAAGGATCCTTAATCCGCGTATGGAGACCAAT	Cloning pS261
SARS_268	GTTCCAGCCAACAGCGCCGTGCTGGCATTTTGCGCGTTCGCAG	Cloning pS262
SARS_269	ACGGCGCTGTTGGCTGGAACTTCGGCGGCATTACCTGCTGCGGCC	Cloning pS262
SARS_270	TAAGGCCTACCTGGCAGCGGGCGGTCAGCCGATTA	Cloning pS263
SARS_271	CCGCTGCCAGGTAGGCCTTATACGCCTTTGCTGCG	Cloning pS263
SARS_272	CAGCGTGCGCGGCCGCAGCGGACGCAGCAAAGGCGTA	Cloning pS264
SARS_273	GCTGCGGCCGCGCACGCTGACAGCACGGTGCTGTTG	Cloning pS264
SARS_280	AGCCAACCTATGGGGTTGGGTACCAGCCTT	Cloning pS267
SARS_281	CCAACCCCATAGGTTGGCTGGAAGCCATAGG	Cloning pS267
SARS_287	CGTTCAAATCTTATGGTGTAAGTCCGACTAAATTGAACG	Cloning pS271
SARS_288	ACTTACACCATAAGATTTGAACGTACTGAAGCTCGCA	Cloning pS271

SARS_289	CAGGATCTGTAATCGCGTGGAATTCTAATAACCTG	Cloning pS272, pS273
SARS_290	CCACGCGATTACAGATCCTGTGAAGTCATCGGGC	Cloning pS272, pS273
SARS_291	GGCGTAAAAGGCTTTAACTGCTATTTCCCATTGC	Cloning pS276, pS278
SARS_292	CAGTTAAAGCCTTTTACGCCATTACACGGTGTGC	Cloning pS276, pS278
SARS_293	GGAACATCGCAGACTATAACTATAAACTGCCCG	Cloning pS277, pS279, pS280
SARS_294	AGTTATAGTCTGCGATGTTCCCTGTTTGCCCAGGAG	Cloning pS277, pS279, pS280
SARS_295	ATAACTACCGTTACCGTCTGTTTCGCAAGTCA	Cloning pS275
SARS_296	CAGACGGTAACGGTAGTTATAGTTGCCTCCAACCT	Cloning pS275

References

1 Deaconescu AM, Chambers AL, Smith AJ, Nickels BE, Hochschild A, Savery NJ & Darst SA (2006) Structural Basis for Bacterial Transcription-Coupled DNA Repair. *Cell* **124**, 507–520.

2 Dove SL, Joung JK & Hochschild A (1997) Activation of prokaryotic transcription through arbitrary protein–protein contacts. *Nature* **386**, 627–630.

3 Deighan P, Diez CM, Leibman M, Hochschild A & Nickels BE (2008) The bacteriophage λ Q antiterminator protein contacts the β-flap domain of RNA polymerase. *Proc Natl Acad Sci* **105**, 15305 LP – 15310.

4 Kuznedelov K, Minakhin L, Niedziela-Majka A, Dove SL, Rogulja D, Nickels BE, Hochschild A, Heyduk T & Severinov K (2002) A role for interaction of the RNA polymerase flap domain with the σ subunit in promoter recognition. *Science (80-)* **295**, 855–857.

5 Vallet-Gely I, Donovan KE, Fang R, Joung JK & Dove SL (2005) Repression of phase-variable cup gene expression by H-NS-like proteins in Pseudomonas aeruginosa. *Proc Natl Acad Sci U S A* **102**, 11082 LP – 11087.
